# Latent profile analysis of knowledge, attitude and practice of hospital infection prevention and control among haemodialysis nurses in Sichuan, China: a multicenter study

**DOI:** 10.3389/fpubh.2026.1734891

**Published:** 2026-02-17

**Authors:** Li He, Wen-Wen Yu, Hao-Tian Zheng, Xu-Hua Zhou, Fu Qiao, Ying-Jun Zhang, Lin Chen

**Affiliations:** 1 Hemodialysis Center, Department of Nephrology, West China Hospital, Sichuan University/West China School of Nursing, Sichuan University, Chengdu, China; 2 Department of Infection Control, West China Hospital, Sichuan University, Chengdu, China

**Keywords:** nurse, haemodialysis, hospital infection, knowledge-attitude-practice, latent profile analysis

## Abstract

**Background:**

Hemodialysis, as one of the main alternative treatment methods for end-stage renal disease, has received much attention in recent years. Due to the particularity of hemodialysis treatment, patients have a relatively high risk of infection during the treatment process. Hemodialysis nurses, who are the main executors of the treatment operations and have the most contact with patients, have a close relationship with the infection risk of patients. The level of their hospital infection prevention and control literacy is closely related to the infection risk of patients.

**Objective:**

To explore the current level of knowledge, attitudes, and practices (KAP) of hospital infection prevention and control among haemodialysis nurses in the Sichuan Province, China, and identified their potential categories. This provided evidence-based recommendations for improving infection control management in hemodialysis departments.

**Methods:**

A cross-sectional study was conducted From July 15 to August 15, 2025 using a convenience sampling method to survey 470 hemodialysis nurses from 78 hospitals in Sichuan Province. Participants were licensed nurses with over 3 months of hemodialysis experience. Data were collected using the *General Information Questionnaire*, the *Haemodialysis Nurses’ KAP Questionnaire on Hospital Infection Prevention and Control* (48 items), the Utrecht Work Engagement Scale-9 (UWES-9) (9 items), and the *Organizational Support Scale* (12 items). Rigorous online data quality control measures were implemented, including IP duplication checks and response time validation. The latent potential profile (LPA) was used to analyze the current level of KAP of hospital infection prevention and control among haemodialysis nurses, and binary Logistic analysis was employed to identify the influencing factors.

**Results:**

A total of 460 valid questionnaires were collected, with an effective response rate of 97.87%. The average scores for knowledge, attitudes, and practices related to hospital infection prevention and control among haemodialysis nurses were 4.67 ± 0.43, 4.59 ± 0.43, and 4.74 ± 0.34, respectively. Three latent profile models were constructed, with the two-class model identified as the optimal solution, which were defined as the “Low KAP Group” (25.9%) and “High KAP Group” (74.1%). Logistic regression analysis revealed that sex, responsibility for infection control, hospital level, annual number of infection control training sessions, organizational support, and work engagement were significant influencing factors (*p* <0.05).

**Conclusion:**

The KAP level of haemodialysis nurses in hospital infection prevention and control was relatively high. Hospital managers should tailor supportive work environments on the basis of the individual characteristics and work engagement of haemodialysis nurses to improve the KAP level of nosocomial infection prevention and control among haemodialysis nurses.

## 1 Introduction

The incidence of chronic kidney disease is high and affects approximately 14.3% of the global population (1). As a major treatment for end-stage renal disease (ESRD), the global population receiving maintenance hemodialysis (MHD) continues to grow, posing significant challenges to healthcare systems worldwide (2). Within Asia, the burden of ESRD is particularly substantial due to demographic transitions and high prevalence of risk factors such as diabetes and hypertension. China, as the most populous country in the region, reflects this trend (3). According to data published in the Chinese National Renal Data System (CNRDS), the total number of patients receiving maintenance haemodialysis (MHD) in China exceeded 100,0000 by the end of 2024. Despite advancements in renal replacement therapy, the survival rate of MHD patients is significantly lower than that of healthy individuals. Owing to immune dysfunction, frequent vascular access punctures of vascular access or indwelling catheters, and underlying diseases in haemodialysis patients, the risk of infection in patients with long-term repeated treatment is significantly increased (4). Infection is among the three main causes of death in MHD patients and its mortality is second only to that of cardiovascular and cerebrovascular diseases (5). Hospital-acquired infections not only worsen the clinical status of haemodialysis patients and increase hospitalization rates and treatment costs (6, 7) but also pose significant threats to patient survival and outcome and may even seriously endanger public health security. The haemodialysis unit is currently the main location where patients undergoing haemodialysis receive regular treatment. The risk for infection increases owing to characteristics such as prolonged coexistence in the same room and frequent personnel movement among haemodialysis patients. Therefore, the haemodialysis unit has always been a key department for hospital infection prevention and control (8). Haemodialysis nurses, as the main practitioners of treatment procedures, have the most direct contact with patients. Their skill in preventing hospital infections is closely related to hospital infection rates (9).

The knowledge-attitude-practice (KAP) theory model is one of the models for changing human health-related behaviors. It divides the process of changing human behavior into three consecutive stages: acquiring knowledge, generating beliefs, and forming behaviors (10). This theory holds that correct health care knowledge and information form the basis for establishing correct and positive beliefs, which in turn lead to changes in health behaviors. The KAP model has been widely applied in various medical fields across different countries. The knowledge, attitudes and practices of nursing staff, as the executors of treatment procedures, are vital for controlling hospital infections. Studies suggest that increased knowledge, attitudes and practices can improve the hand hygiene compliance of nurses (11). A study in South Korea revealed that the correct rate of hospital infection prevention and control knowledge was 87.4%. Nurses demonstrated a positive attitude toward infection prevention and control (7.5/8) and a favorable view of the safety environment (7.75/9), with a compliance score of 91.41. The overall level was relatively high, exceeding 80% in all aspects. However, there is a disparity between the knowledge acquisition of hospital infections and the implementation of behaviors, and true “knowledge and action integration” has not been achieved (12). However, some studies have shown that there are differences between the mastery of knowledge and the implementation of practice, which fail to truly achieve the “unity of knowledge and practice”. Moon JE’s study revealed that while health care workers demonstrated 82.61% accuracy in hospital-acquired infection control knowledge, their behavioral compliance remained suboptimal, with only 33% performing hand hygiene after glove removal (13). Compared with those in other departments, the prevention and control of hospital infections in the haemodialysis unit have their own specific requirements and standards. According to *General standard for infection prevention and control of departments with high infection risk in healthcare facilities WS/T 860—2025*, which was released by the Hospital Infection Control Standard Committee of the National Health Commission of China in 2025 and guided by the committee, it is pointed out that the hemodialysis center is a key department for monitoring infection prevention and control in hospitals (14). In 2023, the National Health Commission of China included the incidence of haemodialysis-related infection in the medical quality control index of hospital infection management (15). The current research on hospital infection prevention and control for medical staff in hemodialysis units lacks a comprehensive investigation into the knowledge, attitude and practice of hemodialysis nurses regarding the overall hospital infection prevention and control. For instance, in the survey conducted by Prevyzi et al. on the prevention and control of hospital infections among hemodialysis healthcare workers, only two aspects knowledge and attitude were examined. The survey results showed that the correct response rate of healthcare workers regarding infection control knowledge varied significantly (ranging from 24.26 to 98.13%) (16). Another survey on the knowledge, attitude and practice of hospital infection prevention and control among hemodialysis nurses in China focused on catheter-related bloodstream infections and did not include an investigation of the overall infection control in hemodialysis (17). Moreover, the published studies mainly employed descriptive and correlational statistical methods. There are no research results available regarding the characteristics of hospital infection prevention and control in this group. Therefore, it is necessary to investigate the knowledge, attitudes and practices of hospital infection prevention and control among hemodialysis nurses, as well as their potential categories, and to analyze the influencing factors. These findings provide a theoretical basis for improving the knowledge, attitudes and practices of haemodialysis nurses, thereby positively affecting the treatment environment of haemodialysis and the prognosis of patients.

## 2 Methods

### 2.1 Study design

This study was a cross-sectional study. From August 1st to August 15th, 2025, we recruited 460 nurses engaged in hemodialysis from 78 hemodialysis centers in Sichuan Province, China.

### 2.2 Participants

The inclusion criteria were as follows: (a) licensed nurses working in haemodialysis units for more than 3 months; and (b) voluntary participation in this study and signing the informed consent form. Nurses on annual leave, sick leave, or maternity leave were excluded. According to the methodological literature on potential profile analysis (18)，we set the target effective sample size to be no less than 400 people to ensure the robustness of the analysis results. This study was approved by the Medical Ethics Committee of West China Hospital, Sichuan University (2025(1259)).

### 2.3 Study tool

This study collected data through a structured online questionnaire, which consisted of the following four sections.

#### 2.3.1 Demographic and occupational characteristics form

A questionnaire was used to collect sociodemographic data, including age, sex, marital status and qualifications as a haemodialysis specialist nurse.

#### 2.3.2 Questionnaire on the knowledge, attitudes and practices of infection prevention and control among haemodialysis nurses in hospitals

Based on domestic and international clinical practice guidelines, expert consensus, and previous literature (19–21), using the knowledge-attitude-practice theory as the framework, we developed a questionnaire on hospital infection prevention and control for hemodialysis nurse. Five experts in hemodialysis and hospital infection fields were invited to revise the questionnaire content through a group meeting, and finally reached a consensus. The expert members include 2 chief physicians, all with doctoral degrees, who have been engaged in haemodialysis and hospital infection control for more than 20 years. One chief nurse, with a bachelor’s degree, has been engaged in haemodialysis for 30 years. There are also 2 supervisor nurses, all with a bachelor’s degree, who have been involved in haemodialysis for more than 10 years. After consulting with experts and conducting discussions, our team revised the questionnaire twice. The questionnaire was pretested on 59 participants, and the results revealed that the Cronbach’s *α* was 0.934, indicating good internal consistency. The final version of the questionnaire is in Chinese (the English translation is provided in Supplementary File 2). The questionnaire is comprised of three dimensions: knowledge, attitudes, and practices, across 48 items. The questionnaire uses a 5-point Likert rating scale. The knowledge dimension has 15 items (Completely understand ~ Completely do not understand), the attitude dimension has 18 items (very agree ~ very disagree), and the behavior dimension has 15 items (completely achievable ~ completely unachievable). Each item is scored from 5 points to 1 point. The total possible score ranges from 48 to 240 points, with higher scores indicating better infection prevention and control competencies. In this study, the Cronbach’s *α* coefficients for the knowledge dimension, attitude dimension and practices dimension were 0.965, 0.934, and 0.922, respectively, while the Cronbach’s α coefficient for the entire questionnaire was 0.966, indicating that the reliability of this questionnaire is good; the KMO value was 0.950, and the Bartlett’s sphericity test *χ*^2^ value was 21572.193, with *p* <0.001, the eigenvalues of the three extracted common factors are all greater than 1, and the cumulative variance contribution rate is 65.753%, suggesting that this questionnaire has good validity.

#### 2.3.3 The Utrecht work engagement scale-9

The measurement of work engagement adopted the simplified version of the Utrecht Work Engagement Scale developed by Schaufeli et al. According to the research conducted by the scale developers, Schaufeli et al., a dimension average score of ≥4 is generally regarded as a reference for a relatively high level of engagement (22). Li et al. adapted and revised the Chinese version of this scale. This scale consists of 9 items and 3 dimensions. Specifically, 3 items pertain to vitality, 3 items relate to dedication, and another 3 items concern concentration. They regard the total score of the scale as a continuous variable for data analysis (23). The simplified version of this scale has a good correlation with the original scale (with a correlation coefficient of 0.92), and the Chinese version has good reliability and validity. This scale uses the Likert 7-point scoring method, with a score ranging from 0 to 54 points. The higher the score on the questionnaire is, the greater the level of work engagement. In this study, for the purpose of conducting inter-group comparisons, we treated the total score of work engagement as a continuous variable based on the knowledge, attitude, and practice (KAP) groups identified through latent profile analysis, and used t-tests/factorial analysis of variance for the comparisons. In this study, the Cronbach’s *α* of the UWES-9 scale was 0.945.

#### 2.3.4 Organizational support scale

The *Organizational Support Scale* compiled by Pan et al. was used. This scale consists of two dimensions, emotional support and instrumental support, with a total of 14 items (24). The Linkert-5 rating scale was adopted. The developers of the scale treated the total score as a continuous variable for processing. The higher the score on the scale is, the greater the sense of organizational support. “The hospital does not care much about my personal feelings” and “The hospital does not care much about my personal development” were scored inversely. The internal consistency of the scale was excellent in this study, with a Cronbach’s *α* of 0.955.

### 2.4 Data collection

The data were collected through the online survey platform “Questionnaire Star platform”.^1^ The chairperson of the Haemodialysis Nursing Professional Committee of the Nursing Society of Sichuan Province, China, assisted in distributing the questionnaires to the participants who met the inclusion criteria. The questionnaires could only be submitted upon complete responses to all the items.

After the data was recovered, we carried out strict data quality control to ensure the validity of the data. Each IP address can only fill out one questionnaire to avoid duplicate responses. The final dataset exported from the platform has completely removed all IP address information. Members of the research team are unable to access or obtain any information related to IP addresses. Therefore, the research team at any stage will not and will not attempt to track or identify any specific participants through IP addresses. If the answers provided by the participants show excessive consistency or contradictory responses, indicating that the participants did not answer seriously and thereby affecting the accuracy of the results, such questionnaires will be regarded as invalid. Based on the test fillings of the questionnaire conducted by the research team and the expert group, it is indicated that the normal time to complete the entire questionnaire is usually more than 10–15 min. Therefore, we will consider any questionnaire completed in less than 10 min as an invalid response.

### 2.5 Statistical analysis

This study employed latent profile analysis to identify different latent categories among hemodialysis nurses in terms of their knowledge, attitude and practice on infection prevention and control. The latent profile analysis was conducted using Mplus 8.3 software. Taking the scores of 48 items in the Questionnaire on the Knowledge, Attitudes and Practices of Infection Prevention and Control among Haemodialysis Nurses in Hospitals as the dependent variables, gradually increase the number of categories in the model until the fitting indicators of the model reach the optimal level. To identify the optimal number of profiles, the following model fit indices were utilized: Akaike Information Criterion (AIC), Bayesian Information Criterion (BIC), Adjusted BIC (aBIC), Lo–Mendell–Rubin Test (LMRT), Bootstrap Likelihood Ratio Test (BLRT), and entropy. Smaller AIC, BIC and aBIC indicated better model fitting. Information X entropy was used to evaluate the classification accuracy of the model. The closer to 1, the higher the accuracy of the model. The likelihood ratio test (Lo–mendell–rubin, LMR) and Bootstrap likelihood ratio test (BLRT) were used to compare the fitting effect of the two adjacent models. *p* <0.05 indicated that the model had a good fitting effect. After the number of latent profiles was determined, data analysis was performed using SPSS 27.0 software. Count data are expressed as frequencies, percentages or proportions, and comparisons between groups were conducted using the *χ*^2^ test. Normally distributed data are expressed as the mean ± standard deviation, whereas nonnormally distributed data are represented as M (P25, P75). The classification results derived from the latent feature analysis were used as the dependent variable. Variables with a *p* value less than 0.05 in the univariate analysis were included as independent variables in the binary Logistic regression analysis. The analysis results were reported in the form of adjusted odds ratios and their 95% confidence intervals. The significance level for the hypothesis test was set at *α* = 0.05.

## 3 Results

### 3.1 Demographic characteristics

A total of 470 haemodialysis nurses completed the questionnaire survey, with 460 valid questionnaires obtained, resulting in an effective response rate of 97.9%. The participant cohort comprised predominantly female nurses (94.8%), with a mean age of 34.74 ± 6.832 years and a median haemodialysis experience of 7.00 (3.00, 12.00) years. Among the respondents, 317 (68.9%) were employed at tertiary hospitals, while 369 (80.2%) had a bachelor’s degree or higher. A history of occupational exposure was reported by 274 nurses (59.6%). These 460 participants were distributed across 78 haemodialysis centers, with individual center representations ranging from 1 to 22 nurses (mean: 6 nurses per center). The detailed demographic characteristics are presented in Table 1.

**Table 1:** Comparison of latent profiles of KAP among HD nurses with different demographic characteristics (*N* = 460).

Variables	Total sample (*n* = 460)	Class 1 (*n* = 119)	Class 2 (*n* = 341)	*χ* ^2^	*p*
Age (years)				2.627	<0.001
≤30	128 (27.8)	52 (43.7)	76 (22.3)		
31–40	245 (53.2)	56 (47.1)	189 (55.4)		
>40	87 (19.0)	11 (9.2)	76 (22.3)		
Gender				13.915	<0.001
Female	436 (94.8)	105 (88.2)	331 (97.1)		
Male	24 (5.2)	14 (11.8)	10 (2.9)		
Marital status				6.831	0.009
Unmarried	80 (17.4)	30 (25.2)	50 (14.7)		
Married	380 (82.6)	89 (74.8)	291 (85.3)		
Education level				3.054	0.217
Junior college	91 (19.8)	30 (25.2)	61 (17.9)		
Undergraduate	362 (78.7)	87 (73.1)	275 (80.6)		
Graduate	2 (1.5)	5 (1.7)	7 (1.5)		
Professional title				16.478	<0.001
Junior	284 (61.7)	92 (78.3)	192 (56.3)		
Intermediate or above	150 (32.6)	27 (22.7)	149 (43.7)		
Working experience (years)				1.359	0.507
1–10	329 (71.5)	105 (88.2)	224 (65.7)		
11–20	120 (26.1)	13 (10.9)	107 (31.4)		
21–40	11 (2.4)	1 (0.9)	10 (2.9)		
History of occupational exposure				0.710	0.400
Yes	274 (59.6)	67 (53.8)	207 (60.7)		
No	186 (40.4)	52 (46.2)	134 (39.3)		
Undertake infection control duties				10.132	<0.001
Yes	176 (38.3)	31 (26.1)	145 (42.5)		
No	284 (61.7)	88 (73.9)	196 (57.5)		
Hospital tiers				25.821	<0.001
Tertiary	317 (68.9)	63 (52.9)	254 (74.5)		
Secondary	81 (17.6)	25 (21.0)	56 (16.4)		
Unclassified	62 (13.5)	31 (26.1)	31 (9.1)		
Annual infection control training sessions attended (times)				7.585	0.006
<5	213 (46.3)	68 (57.1)	145 (42.5)		
≥5	247 (53.7)	51 (42.9)	196 (57.5)		
Organizational support	56.22 ± 10.17	50.66 ± 8.99	58.16 ± 9.84	−7.304	<0.001
Work engagement	41.55 ± 12.51	34.09 ± 14.57	44.16 ± 1.56	−8.062	<0.001

### 3.2 The scores of haemodialysis nurses’ KAP questionnaire on hospital infection prevention and control and the latent profile analysis

The total score of the questionnaire on hospital infection prevention and control among hemodialysis nurses ranged from 48 to 240 points, with an average score of 103.06 ± 19.30 points. The average scores for knowledge items were 4.67 ± 0.43, for attitude items 4.59 ± 0.43, and for behavioral items 4.74 ± 0.34. The details can be found in Table 2. The three items with the lowest score rates among the three dimensions are listed in Table 3.

**Table 2:** Score of questionnaire on KAP of hospital infection prevention and control among hemodialysis nurses.

Dimensions	Number of items	Score range	total points (mean ± SD)	Average item score (mean ± SD)
Knowledge	15	15–75	70.02 ± 6.39	4.67 ± 0.43
Attitude	18	18–90	82.61 ± 7.82	4.59 ± 0.43
Practice	15	15–75	71.14 ± 5.15	4.74 ± 0.34
Total	48	48–240	223.77 ± 16.63	4.66 ± 0.35

**Table 3:** The top 3 items with the lowest scores in each dimension of hospital infection and control KAP.

Dimension	Items	(Mean ± SD)
Three items with the lowest score of knowledge dimension	6. I know the requirements for surveillance of infectious pathogenic microorganisms in hemodialysis patients	4.60 ± 0.60
	10. I know the reporting requirements for patients with emerging infectious diseases in the hemodialysis unit	4.59 ± 0.57
	13. I know the diagnostic criteria for a central venous catheter bloodstream infection	4.58 ± 0.56
Three items with the lowest score of attitude dimension	5. I think the use of protective equipment will affect the accuracy of the operation	4.07 ± 0.88
	12. I think the department infection control training and assessment completion should be included in the performance appraisal system of nurses	4.38 ± 0.82
	13. I am willing to devote extra time and effort to the prevention and control of hospital-acquired infections in the hemodialysis unit.	4.31 ± 0.83
Three items with the lowest score of practice dimension	1. I will regularly check the completion of infectious disease pathogenic microorganism monitoring for hemodialysis patients in my charge	4.34 ± 0.77
	10. When performing the machine connection operation for the patients, I will wear the protective equipment as required.	4.68 ± 0.61
	14. I know how to manage a central venous catheter bloodstream infection	4.70 ± 0.54

**Figure 1:**
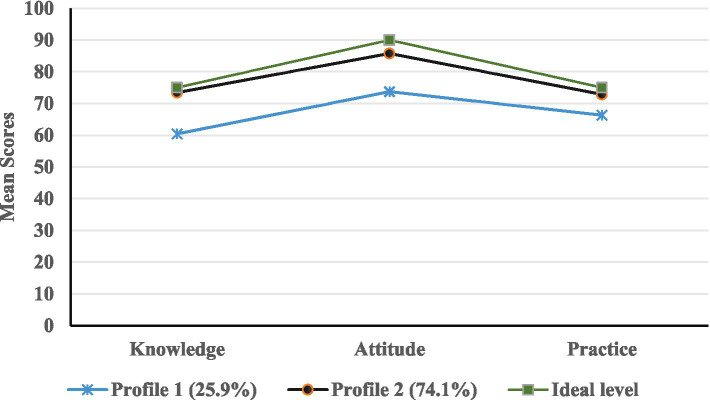
Distribution of characteristics among the two latent profiles of KAP.

The scores for the three dimensions of the KAP questionnaire (knowledge, attitudes, and practices) were analyzed using latent profile analysis (LPA) in Mplus 8.3 software. The models were fitted with 1 to 3 latent profiles. The results of the different model adaptation indicators are shown in Table 4. As the number of profiles increased, the values of AIC, BIC, and aBIC gradually decreased. When 2 profiles were retained, the entropy index score was the highest (0.955), indicating the optimal classification accuracy. Moreover, the LMR and BLRT values were both less than 0.05. Finally, Model 2 was selected as the best fitting model, with the probabilities of each profile being 0.741 and 0.259, respectively. The distribution of potential characteristics is shown in Figure 1. The first category consists of 119 nurses (25.9%), whose scores are very low in all dimensions of knowledge, attitudes and practices, and they are referred to as the “low-KAP group.” The second category consists of 341 nurses (74.1%), whose scores are high in all dimensions, and they are referred to as the “High-KAP group.” The scores of each characteristic in the dimensions of knowledge, attitude and practice are shown in Table 5.

**Table 4:** Latent profile analysis of KAP with fit indices.

Model	AIC	BIC	aBIC	Entropy	LMR	BLRT	Categorical probability
1	9031.888	9056.675	9037.633	–	–	–	–
2	8345.869	8387.182	8355.444	0.955	0.0036	0.0043	0.259/0.741
3	8217.474	8275.312	8230.879	0.937	0.4511	0.4652	0.152/0.711/0.137

AIC, Akaike information criterion; BIC, Bayesian information criterion; aBIC, adjusted Bayesian information criterion; Entropy, Entropy test; LMR, Lo–Mendell–Rubin likelihood ratio test; BLRT, Bootstrapped likelihood ratio test.

**Table 5:** Profiles differences in the KAP and the results of *post hoc* analysis.

Variables	Class 1 (mean±SD)	Class 2 (mean±SD)	*t*	*p*
Knowledge	60.34 ± 3.54	73.40 ± 2.56	−43.172	<0.001
Attitude	73.64 ± 7.82	85.74 ± 4.82	−19.756	<0.001
Practice	66.23 ± 6.10	72.86 ± 3.39	−14.640	<0.001
Total Score	200.20 ± 11.16	232.00 ± 8.24	−32.886	<0.001

### 3.3 Univariate analysis of different KAP potential characteristics

The results revealed statistically significant differences in age, sex, marital status, professional title, infection prevention and control status, hospital grade, number of annual infection control training sessions attended, organizational support, and work commitment between the two potential profiles of haemodialysis nurses (*p* <0.05), as shown in Table 1.

### 3.4 Logistic regression analysis of different potential characteristics of KAP

Taking the two potential profiles as dependent variables (low KAP group = 0; high KAP group = 1), age, sex, marital status, professional title, hospital infection prevention and control status, hospital grade, number of annual hospital infection training sessions, total organizational support score, and total work engagement score were included as independent variables in the binary logistic regression analysis. Independent variable assignment: Age (years), ≤30 = 1, 31–40 = 2, >40 = 3; sex, female = 1, male = 2; marital status, unmarried = 1, married = 2; professional title, junior = 1, intermediate or above = 2; undertake infection control duties, yes = 0, no = 1; hospital tiers, tertiary = 1, secondary = 2, unclassified = 3; annual infection control training sessions attended (times), <5 = 1; and ≥5 = 2. The results revealed that sex, the degree of responsibility for infection prevention and control, hospital grade, the number of annual infection prevention training sessions attended, the total score of organizational support, and work engagement were the factors influencing the knowledge, attitudes and practices of haemodialysis nurses (*p* <0.05), as shown in Table 6.

**Table 6:** Logistic regression of different KAP profiles.

Variables	Class 1 vs. Class 2 (ref: Class 1)				
	B	SE	OR	95% CI	*P*
Age (years)					
≤30			Ref		
31–40	0.251	0.333	1.285	(0.670–2.465)	0.451
>40	0.466	0.528	1.593	(0.466–3.448)	0.378
Gender					
Female			Ref		
Male	−1.306	0.505	0.271	(0.101–0.729)	<0.05
Marital status					
Unmarried			Ref		
Married	−0.010	0.361	0.991	(0.488–2.011)	0.979
Professional title					
Junior			Ref		
Intermediate or above	0.440	0.408	1.552	(0.698–3.454)	0.281
Undertake infection control duties					
Yes			Ref		
No	−0.808	0.356	0.446	(0.222–0.896)	<0.05
Hospital tiers					
Tertiary			Ref		
Secondary	−0.661	0.334	0.516	(0.273–3.340)	<0.05
Unclassified	−1.067	0.372	0.344	(0.273–3.340)	<0.05
Annual infection control training sessions attended (times)					
<5			Ref		
≥5	0.655	0.255	1.924	(1.166–3.175)	<0.05
Organizational support	0.046	0.011	1.047	(1.024–1.070)	<0.001
Work engagement	0.052	0.016	1.053	(1.021–1.086)	<0.001

## 4 Discussion

This study shows that the scores of blood dialysis nurses in terms of knowledge, attitude and practice regarding hospital infection prevention and control are generally at an above-average level. Notably, the score of the attitude dimension regarding hospital infection prevention and control among haemodialysis nurses was the lowest, while the score of the practices dimension was the highest. This is similar to the existing research results (12, 13), suggesting a potential attitude-practice gap in this population. This discrepancy may reflect insufficient awareness among some haemodialysis nurses of the critical role that infection control plays in haemodialysis unit management. They believed that some of the prevention requirements were added to the clinical workload, such as the item with the lowest score in the attitude dimension “I think the use of protective equipment will affect the accuracy of the operation”. In terms of practice, since the haemodialysis unit is a critical department for hospital infection prevention and control, it needs to undergo inspections and supervision at all levels. This structured oversight system effectively reinforces compliance with standardized infection prevention protocols among haemodialysis nursing staff. However, the relative weakness in specific practice items (e.g., When performing the machine connection operation for the patients, I will wear the protective equipment as required) hints at supervision-dependent compliance, where adherence may wane in the absence of direct observation. Therefore, moving beyond knowledge dissemination, intervention strategies should aim to reframe hospital infection prevention and control as a core value rather than a compliance task. *The standards for preventing and controlling hospital infections in hemodialysis centers*, which were formulated by the National Health Commission of China, recommend that hemodialysis medical staff should correctly master the relevant infection prevention and control measures (25). This is crucial for maintaining autonomous compliance and bridging the gap between attitudes and reliable practices.

This study conducted a latent profile analysis on the knowledge, attitude and practice scores of blood dialysis nurses regarding hospital infection prevention and control and divided them into a “low-KAP group” and a “high-KAP group.” The high-KAP group accounted for 74.1% of the participants, demonstrating that the majority of haemodialysis nurses maintained relatively strong competencies in KAP regarding hospitals for infection prevention and control. These findings align with previous research by Ayed et al. (26, 27). The main reason is that the haemodialysis department is key for hospital infection management. As haemodialysis units constitute critical departments for hospital infection control, multiple guidelines and regulations need to be followed at both the national and departmental levels (8). Moreover, regular infection prevention and control training is conducted to enhance the hospital infection prevention skills of nurses to prevent the occurrence of hospital-acquired infections. Even in such an environment, 25.87% of the nurses still had a low level of knowledge, attitudes and practices. Therefore, it is necessary to pay attention to the specific situations of these nurses and provide them with targeted training.

This study revealed that female haemodialysis nurses are more likely to be in the high-KAP group, which is similar to findings from foreign research (26, 28). This might be related to the high percentage of female nurses in the haemodialysis unit in this study (94.8%). Female nursing staff in the haemodialysis unit perform more tasks related to infection prevention and control. Nurses who were responsible for infection prevention and control were more likely to be in the high-KAP group. Their managerial roles necessitate greater exposure to updated infection prevention and control guidelines, regular participation in hospital-level infection prevention and control committees, and ongoing professional training (29). This continuous engagement fosters a deeper understanding of infection prevention and control principles and cultivates a more positive attitude toward control measures (30). As a result, these nurses maintain a positive attitude toward hospital infection control measures. As the managers of the department, the behaviors of the nurses who are responsible for hospital infection control become the standard for the daily work of other nurses, which in turn promotes the standardized behavior of the nurses who are responsible for hospital infection control. Therefore, in clinical practice, strengthening training for general medical staff in haemodialysis units, enhancing their understanding of the importance of infection prevention and control, and thereby improving their knowledge, attitudes and practices are necessary.

This study revealed that nurses in tertiary hospitals were more likely to be included in the high-KAP group, which is consistent with the findings of Liu et al. (31). This phenomenon may be attributed to the fact that tertiary hospitals are the highest among hospitals in China, and such hospitals have a complete allocation of infection prevention and control resources as well as an established infection prevention and control management system. Moreover, these hospitals have developed systematic training programs. Prior to assuming clinical duties, nurses must pass rigorous infection control knowledge assessments, with specialized training mandated for high-risk haemodialysis procedures. In China, the qualification assessment for the dedicated infection control personnel in tertiary hospitals (78.89%) and the frequency of regular training (43.75% of which is monthly training) are both at a relatively high level (32). In addition, the departments of these hospitals need to establish standardized operation procedures, emphasizing the standardization of operational details. Daily supervision and assessment also play a promoting role in the behavioral norms of medical staff.

Studies have shown that compared with those who did not receive training, nurses who received training had greater levels of knowledge, attitudes and practices regarding hospital infection prevention and control. The existing surveys have mentioned that monthly training and quarterly training are currently the most common training frequencies in China’s tertiary hospitals. However, similar to studies in other countries, the specific number of training courses has not been explored (32, 33). Owing to the critical importance and unique challenges of infection control in haemodialysis settings, all haemodialysis nurses are required to undergo infection control training. However, whether more training sessions lead to better results is a question worth discussing. This study revealed that nurses with fewer than 5 training sessions were more likely to be in the high-KAP group. This phenomenon may be attributed to the appropriate number of training sessions not causing information overload for nurses. While more training sessions do not enable nurses to absorb and transform their knowledge. Information overload can easily lead to negative emotions such as tension and stress among medical staff, making it not only difficult for nurses to focus on learning core knowledge, but also likely to cause problems such as extended working hours and improper decision-making (34, 35). Therefore, hospital managers should consider the appropriateness of the training format and frequency when designing infection prevention and control training programs for haemodialysis nurses to enhance the knowledge, attitudes and practices related to infection prevention and control among haemodialysis nurses.

Nurses with higher levels of organizational support and greater job involvement are more likely to be classified into the high-KAP group. These findings align with established evidence that organizational support is positively correlated with nursing staff engagement levels (36, 37). When nurses perceive adequate organizational support, they develop a stronger sense of organizational commitment. This positive psychological state enhances work engagement, manifested through increased vigor, focus, and professional dedication (38). High levels of participation can motivate nurses to study, understand and apply knowledge related to infection prevention and control more actively, thereby enabling them to have a deeper understanding of its importance. Moreover, in the face of complex haemodialysis procedures and high infection-risk environments, they can more accurately implement various standardized infection control protocols. These findings suggest that hospital managers not only need to provide training in knowledge and skills for nurses but also need to create a supportive working environment to stimulate their intrinsic enthusiasm and sense of responsibility, thereby achieving the unified and effective transformation of knowledge, attitudes and practices (39).

This study adopted convenience sampling, which may lead to selection bias. The majority of the research subjects included in the study were from tertiary hospitals in China, indicating that the knowledge, attitude, and practice levels of the selected subjects were relatively high. This might result in an overly high overall KAP rate, thereby affecting the estimation proportion of the potential characteristic groups to a certain extent. However, the identification of these two different potential categories has confirmed that there are significant differences within the study group. Therefore, this finding still has reference value for clinical practice.

## 5 Limitations

First, only nurses from haemodialysis units in Sichuan Province, China, were selected as the survey subjects. This does not represent the overall level of knowledge, attitudes, and practices regarding hospital infection prevention and control among all nurses in haemodialysis units across China. Second，The convenience sampling method used in this study has limitations that affect the representativeness of the research and increase the risk of bias. Future research can expand the scope of investigation and increase the sample size, and adopt methods such as stratified random sampling or multi-stage cluster sampling, in order to more comprehensively understand the overall knowledge level, attitudes and practices of nurses in hemodialysis wards regarding hospital infection prevention and control, as well as the related influencing factors. Finally, this study employed self-reporting methods to assess infection prevention practices. The self-assessment questionnaire provided a feasible and effective means to achieve the goals of this study and to conduct potential profile classification. Future research could attempt to correlate the different profiles identified in this study with their corresponding objective clinical practice indicators in order to further validate the external validity of these profiles.

## 6 Clinical and practical implications

This study shows that in the knowledge, attitude, and practice of hospital infection prevention among hemodialysis nurses, the “low KAP” group of nurses was significantly associated with less training, less sense of organizational support, and lower job engagement. The “high KAP” group performed better than the “low KAP” group in all dimensions of knowledge, attitude, and practice. In clinical practice, we can identify the “low KAP” group early and provide them with re-training and comprehensive management. At the same time, we can also adopt an intervention approach where the “high KAP” group guides the “low KAP” group.

## 7 Conclusion

The overall knowledge, attitude and practice of hemodialysis nurses in Sichuan Province, China, regarding hospital infection prevention and control are relatively high. In this study, the knowledge, attitude and practice of hemodialysis nurses in terms of hospital infection prevention and control can be classified into two potential categories: “High KAP group” and “Low KAP group.” Among them, gender, whether undertaking hospital infection control duties, hospital grade, and the number of times participating in hospital infection prevention and control training are all factors influencing the knowledge, attitude and practice of hemodialysis nurses regarding hospital infection prevention and control. Hospital administrators should implement targeted training measures based on the individual characteristics of medical staff, and provide supportive working environments to enhance the knowledge, attitudes and practice of hospital hemodialysis medical staff in infection prevention and control.

## Data Availability

The original contributions presented in the study are included in the article/Supplementary material, further inquiries can be directed to the corresponding authors.

## Footnotes

1
https://www.wjx.cn/
