# Supplementary material for: Latent profile analysis of knowledge, attitude and practice of hospital infection prevention and control among haemodialysis nurses in Sichuan, China: a multicenter study
Source: Front Public Health. 2026 Feb 17;14:1734891. doi: 10.3389/fpubh.2026.1734891 (PMC12955735; doi:10.3389/fpubh.2026.1734891)
Supplement: Supplementary file 1 [file Data_Sheet_1.docx]

**Knowledge, Attitude and Practice of Hospital Infection Prevention and Control among Hemodialysis Nurses**

**Knowledge**

*Regarding the knowledge of hospital infection prevention and control, please choose the appropriate option according to your real cognition.*

| Items | Completely understand | Understand | Uncertain | Do not understand | Completely do not understand |
| --- | --- | --- | --- | --- | --- |
| 1. I know the specific content of "three zones and two channels" in hemodialysis units |  |  |  |  |  |
| 1. I know the examination items that need to be conducted for patients undergoing hemodialysis before their first dialysis treatment |  |  |  |  |  |
| 1. I know the disinfection requirements of objects and environment after hemodialysis treatment |  |  |  |  |  |
| 1. I know the process of medical waste disposal in the hemodialysis units |  |  |  |  |  |
| 1. I known the monitoring requirements for object surfaces and air in the hemodialysis units |  |  |  |  |  |
| 1. I know the requirements for surveillance of infectious pathogenic microorganisms in hemodialysis patients |  |  |  |  |  |
| 1. I know the criteria for isolation and release of infectious diseases in hemodialysis patients |  |  |  |  |  |
| 1. I known the monitoring requirements for dialysis water and dialysis fluid |  |  |  |  |  |
| 1. I know the reporting requirements for facility-acquired infections occurring in hemodialysis units |  |  |  |  |  |
| 1. I know the reporting requirements for patients with emerging infectious diseases in the hemodialysis unit |  |  |  |  |  |
| 1. I know the timing and requirements of hand hygiene in hemodialysis treatment |  |  |  |  |  |
| 1. I know when to wear gloves during hemodialysis therapy |  |  |  |  |  |
| 13.I know the diagnostic criteria for a central venous catheter bloodstream infection |  |  |  |  |  |
| 14.I know how to manage a central venous catheter bloodstream infection |  |  |  |  |  |
| 15.I know the handling and reporting process of occupational exposure events in hemodialysis units |  |  |  |  |  |

**Attitude**

*Regarding the attitude of hospital infection prevention and control, please choose the appropriate option according to your true willingness*

| Items | Very agree | Agree | Uncertain | Disagree | Very disagree |
| --- | --- | --- | --- | --- | --- |
| 1.I think nursing staff need to comply with the laws and regulations related to hospital infection |  |  |  |  |  |
| 1. I think nursing staff need to be aware of the characteristics of nosocomial infections associated with hemodialysis |  |  |  |  |  |
| 3.I think the hemodialysis unit should carry out the monitoring of nosocomial infection and report it according to the requirements of the hospital |  |  |  |  |  |
| 4.I think the drying time of disinfectant during hemodialysis operation will directly affect the disinfection effect |  |  |  |  |  |
| 5.I think the use of protective equipment will affect the accuracy of the operation |  |  |  |  |  |
| 6.I think that central venous catheter-related infections are largely preventable |  |  |  |  |  |
| 1. I think that timely reporting of occupational exposures is important |  |  |  |  |  |
| 1. I think the classification and management of medical waste is very important |  |  |  |  |  |
| 1. I think that nursing staff should actively participate in the training of knowledge and skills related to hospital infection management |  |  |  |  |  |
| 1. I think that the risk of nosocomial infection is higher in the hemodialysis unit than in the general unit |  |  |  |  |  |
| 1. Compared with part-time hospital infection nurses, I think the hemodialysis unit should have full-time hospital infection nurses to carry out infection control work |  |  |  |  |  |
| 1. I think the department infection control training and assessment completion should be included in the performance appraisal system of nurses |  |  |  |  |  |
| 1. I am willing to devote extra time and effort to the prevention and control of hospital-acquired infections in the hemodialysis unit. |  |  |  |  |  |
| 1. I am willing to take the initiative to learn relevant standards or guidelines for infection control |  |  |  |  |  |
| 1. I am willing to implement the relevant requirements in strict accordance with the hospital infection management standards |  |  |  |  |  |
| 1. I am willing to perform the cleaning and disinfection of the hemodialysis machine according to the standard |  |  |  |  |  |
| 1. I am willing to follow the seven steps of hand hygiene when indicated |  |  |  |  |  |
| 1. I would be willing to isolate patients differently depending on the route of transmission |  |  |  |  |  |

**Practice**

*Regarding the behavior of nosocomial infection prevention and control, please choose the appropriate option according to your actual situation.*

| Items | Completely achievable | Mostly achievable | Partially achievable | Occasionally achievable | Completely unachievable |
| --- | --- | --- | --- | --- | --- |
| 1. I can take the initiative to consult hospital infection prevention guidelines/ manuals when my work requires |  |  |  |  |  |
| 1. In the event of a nosocomial infection outbreak, I can promptly report to my superiors or the hospital infection management department |  |  |  |  |  |
| 1. When engaged in aseptic technical diagnosis and treatment operations such as arteriovenous fistula puncture and central venous catheter dressing change, I will strictly follow the aseptic technical operation rules |  |  |  |  |  |
| 1. I will disinfect and clean the dialysis machine as required and keep proper records |  |  |  |  |  |
| 1. I can completely avoid reuse of disposable sterile items, regardless of their value |  |  |  |  |  |
| 1. I will regularly check the completion of infectious disease pathogenic microorganism monitoring for hemodialysis patients in my charge |  |  |  |  |  |
| 1. I wear sterile gloves when touching patient mucous membranes or broken skin |  |  |  |  |  |
| 1. I wash my hands before touching a patient, before cleaning or aseptic procedures, and after touching a patient and exposure to the patient's surroundings and bodily fluids |  |  |  |  |  |
| 1. I wash my hands or use quick-drying hand sanitizer when entering different units or cleaning different machines. |  |  |  |  |  |
| 1. When performing the machine connection operation for the patients, I will wear the protective equipment as required. |  |  |  |  |  |
| 1. I will use proper isolation protection |  |  |  |  |  |
| 1. When I have occupational exposure, I will deal with it in time and report it according to the requirements and procedures |  |  |  |  |  |
| 1. I can properly dispose of the medical waste generated during hemodialysis treatment |  |  |  |  |  |
| 1. Every time, I would participate in the relevant knowledge training of hospital infection prevention and control organized by the department |  |  |  |  |  |
| 1. I can complete or cooperate with the monitoring of dialysis water, dialysate, surface and environment |  |  |  |  |  |
