# Supplementary material for: Latent profile analysis of knowledge, attitude and practice of hospital infection prevention and control among haemodialysis nurses in Sichuan, China: a multicenter study
Source: Front Public Health. 2026 Feb 17;14:1734891. doi: 10.3389/fpubh.2026.1734891 (PMC12955735; doi:10.3389/fpubh.2026.1734891)
Supplement: Supplementary file 2 [file Data_Sheet_2.docx]

**Knowledge, Attitude and Practice of Hospital Infection Prevention and Control among Hemodialysis Nurses**

**Knowledge** **dimension score（n=460）**

| Items | Mean±SD |
| --- | --- |
| 1. I know the specific content of "three zones and two channels" in hemodialysis units | 4.62±5.67 |
| 1. I know the examination items that need to be conducted for patients undergoing hemodialysis before their first dialysis treatment | 4.73±0.47 |
| 1. I know the disinfection requirements of objects and environment after hemodialysis treatment | 4.76±0.43 |
| 1. I know the process of medical waste disposal in the hemodialysis units | 4.74±0.47 |
| 1. I known the monitoring requirements for object surfaces and air in the hemodialysis units | 4.68±0.51 |
| 1. I know the requirements for surveillance of infectious pathogenic microorganisms in hemodialysis patients | 4.60±0.60 |
| 1. I know the criteria for isolation and release of infectious diseases in hemodialysis patients | 4.62±0.57 |
| 1. I known the monitoring requirements for dialysis water and dialysis fluid | 4.63±0.56 |
| 1. I know the reporting requirements for facility-acquired infections occurring in hemodialysis units | 4.60±0.56 |
| 1. I know the reporting requirements for patients with emerging infectious diseases in the hemodialysis unit | 4.59±0.57 |
| 1. I know the timing and requirements of hand hygiene in hemodialysis treatment | 4.77±0.44 |
| 1. I know when to wear gloves during hemodialysis therapy | 4.79±0.42 |
| 13.I know the diagnostic criteria for a central venous catheter bloodstream infection | 4.58±0.56 |
| 14.I know how to manage a central venous catheter bloodstream infection | 4.61±0.54 |
| 15.I know the handling and reporting process of occupational exposure events in hemodialysis units | 4.72±0.49 |
| Total | 70.02±6.39 |

**Attitude** **dimension score（n=460）**

| Items | Mean±SD |
| --- | --- |
| 1.I think nursing staff need to comply with the laws and regulations related to hospital infection | 4.76±0.50 |
| 1. I think nursing staff need to be aware of the characteristics of nosocomial infections associated with hemodialysis | 4.76±0.50 |
| 3.I think the hemodialysis unit should carry out the monitoring of nosocomial infection and report it according to the requirements of the hospital | 4.76±0.52 |
| 4.I think the drying time of disinfectant during hemodialysis operation will directly affect the disinfection effect | 4.43±0.88 |
| 5.I think the use of protective equipment will affect the accuracy of the operation | 4.07±0.88 |
| 6.I think that central venous catheter-related infections are largely preventable | 4.49±0.65 |
| 1. I think that timely reporting of occupational exposures is important | 4.47±0.54 |
| 1. I think the classification and management of medical waste is very important | 4.73±0.53 |
| 1. I think that nursing staff should actively participate in the training of knowledge and skills related to hospital infection management | 4.71±0.50 |
| 1. I think that the risk of nosocomial infection is higher in the hemodialysis unit than in the general unit | 4.56±0.77 |
| 1. Compared with part-time hospital infection nurses, I think the hemodialysis unit should have full-time hospital infection nurses to carry out infection control work | 4.63±0.61 |
| 1. I think the department infection control training and assessment completion should be included in the performance appraisal system of nurses | 4.38±0.82 |
| 1. I am willing to devote extra time and effort to the prevention and control of hospital-acquired infections in the hemodialysis unit. | 4.31±0.83 |
| 1. I am willing to take the initiative to learn relevant standards or guidelines for infection control | 4.55±0.61 |
| 1. I am willing to implement the relevant requirements in strict accordance with the hospital infection management standards | 4.64±054 |
| 1. I am willing to perform the cleaning and disinfection of the hemodialysis machine according to the standard | 4.68±0.50 |
| 1. I am willing to follow the seven steps of hand hygiene when indicated | 4.72±0.48 |
| 1. I would be willing to isolate patients differently depending on the route of transmission | 4.72±0.48 |
| Total | 82.61±7.62 |

**Practice** **dimension score（n=460）**

| Items | Mean±SD |
| --- | --- |
| 1. I can take the initiative to consult hospital infection prevention guidelines/ manuals when my work requires | 4.34±0.77 |
| 1. In the event of a nosocomial infection outbreak, I can promptly report to my superiors or the hospital infection management department | 4.76±0.47 |
| 1. When engaged in aseptic technical diagnosis and treatment operations such as arteriovenous fistula puncture and central venous catheter dressing change, I will strictly follow the aseptic technical operation rules | 4.75±0.45 |
| 1. I will disinfect and clean the dialysis machine as required and keep proper records | 4.82±0.40 |
| 1. I can completely avoid reuse of disposable sterile items, regardless of their value | 4.86±0.36 |
| 1. I will regularly check the completion of infectious disease pathogenic microorganism monitoring for hemodialysis patients in my charge | 4.64±0.64 |
| 1. I wear sterile gloves when touching patient mucous membranes or broken skin | 4.84±0.39 |
| 1. I wash my hands before touching a patient, before cleaning or aseptic procedures, and after touching a patient and exposure to the patient's surroundings and bodily fluids | 4.79±0.43 |
| 1. I wash my hands or use quick-drying hand sanitizer when entering different units or cleaning different machines. | 4.76±0.45 |
| 1. When performing the machine connection operation for the patients, I will wear the protective equipment as required. | 4.68±0.61 |
| 1. I will use proper isolation protection | 4.76±0.50 |
| 1. When I have occupational exposure, I will deal with it in time and report it according to the requirements and procedures | 4.85±0.37 |
| 1. I can properly dispose of the medical waste generated during hemodialysis treatment | 4.84±0.39 |
| 1. Every time, I would participate in the relevant knowledge training of hospital infection prevention and control organized by the department | 4.70±0.54 |
| 1. I can complete or cooperate with the monitoring of dialysis water, dialysate, surface and environment | 4.77±0.50 |
| Total | 71.14±5.15 |
